# Finding karstic caves and rockshelters in the Inner Asian mountain corridor using predictive modelling and field survey

**DOI:** 10.1371/journal.pone.0245170

**Published:** 2021-01-20

**Authors:** Patrick Cuthbertson, Tobias Ullmann, Christian Büdel, Aristeidis Varis, Abay Namen, Reimar Seltmann, Denné Reed, Zhaken Taimagambetov, Radu Iovita

**Affiliations:** 1 Department of Early Prehistory and Quaternary Ecology, Eberhard Karls University of Tübingen, Tübingen, Germany; 2 Institute of Geography and Geology, University of Würzburg, Würzburg, Germany; 3 Institute for Archaeological Sciences (INA), Eberhard Karls University of Tübingen, Tübingen, Germany; 4 Faculty of History, Archaeology, and Ethnology, Department of Archaeology, Ethnology, and Museology, Al Farabi Kazakh National University, Almaty, Kazakhstan; 5 Department of Earth Sciences, Centre for Russian and Central EurAsian Mineral Studies, Natural History Museum, London, United Kingdom; 6 Department of Anthropology, University of Texas, Austin, United States of America; 7 National Museum of the Republic of Kazakhstan, Nur-Sultan, Kazakhstan; 8 Department of Anthropology, Center for the Study of Human Origins, New York University, New York, United States of America; Universita degli Studi di Milano, ITALY

## Abstract

The area of the Inner Asian Mountain Corridor (IAMC) follows the foothills and piedmont zones around the northern limits of Asia’s interior mountains, connecting two important areas for human evolution: the Fergana valley and the Siberian Altai. Prior research has suggested the IAMC may have provided an area of connected *refugia* from harsh climates during the Pleistocene. To date, this region contains very few secure, dateable Pleistocene sites, but its widely available carbonate units present an opportunity for discovering cave sites, which generally preserve longer sequences and organic remains. Here we present two models for predicting karstic cave and rockshelter features in the Kazakh portion of the IAMC. The 2018 model used a combination of lithological data and unsupervised landform classification, while the 2019 model used feature locations from the results of our 2017–2018 field surveys in a supervised classification using a minimum-distance classifier and morphometric features derived from the ASTER digital elevation model (DEM). We present the results of two seasons of survey using two iterations of the karstic cave models (2018 and 2019), and evaluate their performance during survey. In total, we identified 105 cave and rockshelter features from 2017–2019. We conclude that this model-led approach significantly reduces the target area for foot survey.

## 1. Introduction

Central Asia is one of the emerging hotspots for human evolution research. Recent finds have suggested that at least three metapopulations, the Neanderthals, modern humans, and the newly discovered Denisovans overlapped [1–5] in this part of the world for tens of thousands of years, likely influencing the makeup and structure of contemporary Asian populations [6]. So far, the most important fossil and archaeological discoveries have come from western central Asia [7] and the Altai region in Russia [8]. However, a complete understanding of Late Pleistocene hominin dispersals is not possible without a thorough investigation of the area connecting these two regions [9–11]. In particular, the piedmont areas flanked by the high mountain and lowland deserts are considered a likely location for Pleistocene *refugia* and might have functioned as an ‘Inner Asian Mountain Corridor’ (IAMC, [12]) for dispersal. Yet, so far, most of the Pleistocene archaeology found in the IAMC consists of undated surface sites and open-air sites with relatively short chronologies [13–15, see 16 for a review]. Trends in the currently available data suggest that cave and rockshelter contexts might provide the long sequences needed to begin reconstructing the wider picture of hominin dispersal in the region [9]. Caves and rockshelters have several advantages in comparison with open air sites, in that they can function simultaneously as sediment traps [17] and stable landscape attractors for humans and animals alike. They can provide exceptional records of environmental [18] and archaeological material [19], in case good preservation conditions are present. There is also the possibility of speleothems and vertebrate remains to contribute to palaeoenvironmental reconstruction. Sequences provided by caves can provide an element of chronological control and environmental information that is often absent from open air sites [17,20]. Cave sediments have even provided ancient DNA evidence of human occupation [21].

Around 47% (ca. 211,500km²) of the area of the IAMC is within the modern territory of Kazakhstan alone, making it a prime study region for research questions relating to hominin occupation. However, only two cave sites with probable Pleistocene archaeology were published before: Peshchera (now submerged) in East Kazakhstan [22] and Ushbas in South Kazakhstan [23]. Another prominent cave, also in South Kazakhstan, is Qaraungir (Karaungur), but it has only yielded Holocene (Neolithic) archaeology [24]. Moreover, detailed speleological maps with cave locations are missing for the majority of the karst deposits in Kazakhstan [25,26]. The paucity of available data means that cave sites would have to be discovered by survey. However, the challenge of surveying such a large region requires us to reduce the potential survey area to provide a realistic and targeted approach, and to use our resources most effectively. Moreover, traditional predictive modelling approaches, where a large sample of existing site data are used to predict the likely location of undiscovered sites [27] cannot be used, due to the small sample size of sites initially available. Here we present the results of two predictive models using landform classification, where the results of an initial unsupervised model are used to structure a foot survey, and the results of this survey are used to inform a second model based on supervised classification.

## 2. Study areas

**Fig 1:**
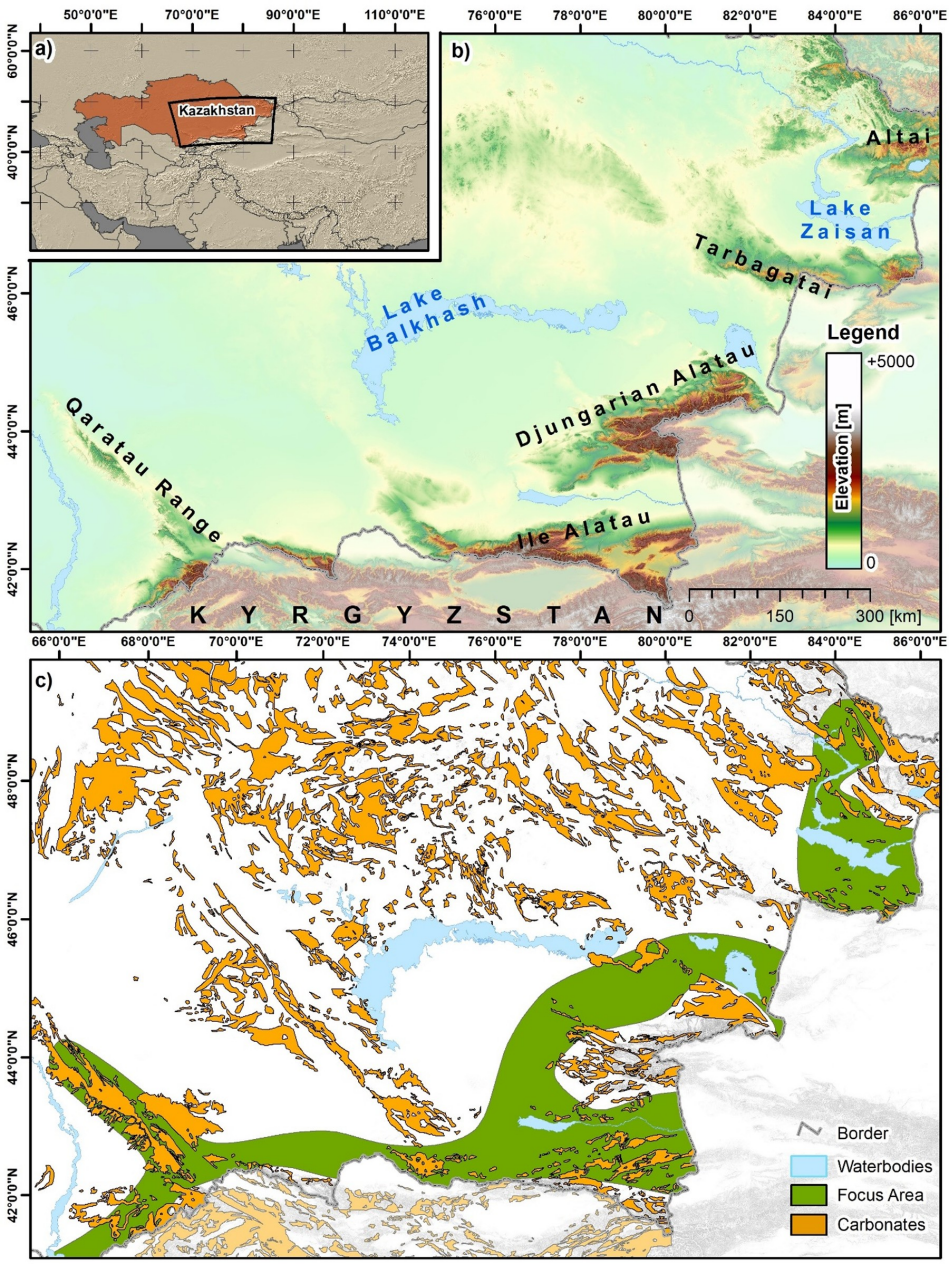
Location, topographic and geological setting of the study area. (a) Location of the study area, (b) Terrain Elevation from the ASTER Digital Elevation Model (DEM) and (c) spatial distribution of formations containing carbonate rock [28], and the focus area of the IAMC. UTM Zone 44N, WGS 1984 ellipsoid (EPSG: 32644). Contains data from ASTER GDEM2 (see section 3.4 for full information). Administrative boundaries and waterbodies use copyrighted map data from OpenStreetMap contributors [29], available from *openstreetmap*.*org*.

Our four key study regions target the extent of carbonate deposits found in the foothill and piedmont zones of southern and southeastern Kazakhstan (see Fig 1), an area of the IAMC.

### 2.1 Qaratau range

The Qaratau mountain range in southern Kazakhstan has developed along the north-western edge of the Talas-Fergana fault, and is therefore related to the Tien Shan through the Talas and Fergana ranges. The Qaratau range is sometimes divided into a western ‘greater’ half and an eastern ‘lesser’ range, which are separated by some 25km in Baydibekskiy Rayon. The range is bordered on either side by the Qyzylkum, Betpaqdala, and Muyunkum deserts. A large number of river valleys wind from the interior of the range out towards the plains, providing sheltered areas of increased vegetation with both seasonal and perennial water sources. The topographic expression of the Qaratau range allows it to act as a sediment trap in an area that is otherwise prone to deflation. This can be seen in the thickness of the Quaternary deposits in the region, which range in thickness from negligible (deflated) up to around 110m in some areas. The Qaratau range has a rich structural history with multiple deformation events and major strike slip faults [30]. The carbonates in the range constitute a platform (*c*. 4km thick), formed during the Late Devonian and Carboniferous with a wide variety of distinctive facies ranging from tidal-flat to deep-water deposits [31]. An increased rate of uplift since the Late Pliocene-Quaternary [32,33] has resulted in the exposure of the carbonate sequence in this area. Due to its proximity to notable Pleistocene cave sites in Uzbekistan (Obi-Rakhmat [34], Teshik Tash [35], Anghilak [36], Dodekatym [37]) and Kyrgyzstan (Sel’ungur [38]), we extended our study region southwards to include the area of Sairam-Ugam.

### 2.2 Ili Alatau

The Ili Alatau is a northern spur of the Tien Shan range. Our study region here includes the Ili depression, bordered to the north by the Borohoro mountains, and to the south by the Tien Shan. Substantial loess deposition has taken place against the foothills of this region. Thickness of the Quaternary deposits in the region is up to 700m in areas with substantial deposition. Along with the ‘Dzhungar gates’, this area represents one possible route of access for Pleistocene hominins between Kazakhstan and northwestern China.

### 2.3 Dzhungarian Alatau

The so-called ‘Dzhungar gates’ represent a narrowing of the landscape to the southeast of Lake Alakol, leading into the Dzhungarian Basin at the modern border of Kazakhstan and China. The flat, deflated area of the ‘gates’ is predominantly arid and windswept, and is constrained by the more humid, vegetated foothills and mountainous areas of the Dzhungarian Alatau. It provides both a mode of egress through the mountain range, as well as a possible ‘bottleneck’ for movement between modern Kazakhstan and China. From this perspective, the area is particularly pertinent for studying possible hominin movement through this region of Asia during the Pleistocene.

### 2.4 Altai-Tarbagatai

The Altai mountains are shared between four countries (Russia, China, Mongolia, Kazakhstan), with its southwestern-most extent stretching into the east of Kazakhstan. Our northern-most study region is constrained by the Kazakh portion of the Altai mountains to the north, and to the south by the Tarbagatai range, centred around the Zaisan basin, through which the Irtysh river flows. Due to its higher latitude, it should be expected that climatic conditions in the Kazakh Altai would have been especially harsh compared with those in our other study areas. The proximity of this study region to the Russian Altai sites make it particularly interesting, as does the presence of the open-air site of Ushbulaq to the south of the Zaisan Basin [15].

All four regions contain formations with carbonate deposits [28]. From Fig 1B, it can be seen that the extent of carbonate deposits includes, but is not limited to, mountainous areas and the areas of adjacent foothills. Where carbonate deposits and karstic systems may become exposed in areas of complex topography, especially within the area of the IAMC, is a key factor structuring the PSR project’s approach.

## 3. Methods and data

### 3.1. Predictive modelling

In the present archaeological literature, there are several published predictive models that are especially relevant to the present study. Beeton et al. [39] and Glantz et al. [40] both look at site distribution in the area of the IAMC in relation to abiotic ecological variables, from which they derive some important conclusions for hominin occupation in our study region. The model produced by Märker & Heydari-Guran [27] is also relevant, as they use a DEM for the identification of caves through landform classification in Iran, which is similar to our own goals and the methods developed here.

Beeton et al. [39] used ecological niche modelling to examine the relationship between late Pleistocene site location and abiotic variables derived from Last Interglacial (LIG) and Last Glacial Maximum (LGM) climate models. From their analyses, the authors concluded that late Pleistocene site location appears aggregated in the area of the IAMC during both the LIG and the LGM. Low temperatures seem to be the chief constraint on the area of hominin occupation during glacial periods, with the foothills of the IAMC provided an apparent string of *refugia*. Glantz et al. [40] followed this study by extending their modelling to include open areas of steppe and steppe-desert adjacent to the IAMC with an ecological threshold model focused on four abiotic variables. They concluded that the foothill zones of the IAMC provided a richer and more attractive environment for hominins during both glacial and interglacial periods, and that this contrast was most extreme during interglacials. Both of these studies together suggest that the area of the IAMC is likely to have provided a core area for hominin occupation in the region throughout the Pleistocene.

Märker & Heydari-Guran [27] used topographic indices derived from a 90m resolution Shuttle Radar Topography Mission (SRTM) DEM, to examine the relationship of Palaeolithic site location to local geomorphology in the Zagros mountains (Iran). Their analysis suggests a relationship between site location and topographic indices such as curvature and slope. They extended this with a random forest model based (i.e. a non-parametric machine learning approach) on these indices, producing a predictive surface for Palaeolithic site location across their study region. This study has provided a very effective proof of concept for using topographic indices for predictive modelling of Palaeolithic sites, but ground-truthing of the model, if it has been undertaken, is not currently published. Furthermore, an integration of predictive modelling and field surveys, with the purpose of validating and extending model prediction results and data interpretation, has not previously been attempted at these scales of analysis.

The morphology of karstic landscapes can be quite specific depending upon climate, lithology, and structure [41]. Geomorphological studies of karst landforms in semi-arid regions are limited (for instance, see [42] for an example of arid and semi-arid areas), while scarce information is available for the area of East Kazakhstan. However, thick carbonate deposits should provide potential for cave formation. In this regard, Heydari [43] has observed that the majority of the Palaeolithic occupied caves and rockshelters in Iran come from an area he defines as the ‘Massive Karstic Mountain System’ zone, a system of uplifted, massive limestone, karstic in expression and dissected by drainage systems.

Having information on the surface morphology and on the extent and nature of deposits that could support karstic features, predictive models can be produced that reduce the possible survey area for a more targeted survey approach. The production of such models is reliant on two sources of data. Firstly, it requires a spatial extent of carbonate geologies in which karstic features can form. Secondly, it requires a digital representation of the surface morphology, e.g. DEM, to characterize the surface morphology, the topographic setting respectively. If an unsupervised method of landform classification is used, then it becomes possible to identify novel areas of potential karstic development, without relying on known location of extant karstic features in the study region. This has two advantages, in that the model is not limited by the known record (which may be a small or unrepresentative sample), and it also requires less data *a priori* to produce. Both of these advantages make an unsupervised model the best choice for the first model prior to systematic survey.

When the location of a substantial number of cave and rockshelter features in the study region is known, supervised kinds of landform classification become more tenable. It is then possible to build a classification model that takes the known locations of extant karstic features, and uses their relationship to other spatial datasets (such as features derived from a DEM) to predict the probability of similar features being present across the study region.

We built two models, one of the former unsupervised type and one of the latter supervised type, to guide survey during the 2018 and 2019 field seasons respectively (details are provided in Section “*3*.*5*. *The 2018 model*” and “*3*.*6*. *The 2019 model*”). Because the models relate directly to the fieldwork goals of the project, our researchers also needed access to the model in the field for orientation and ground truthing, and some form of satellite navigation system for ease of navigating in relation to the model. This allowed a new, considerably advanced degree of model integration into to the field survey strategy and the overall study design.

A common way to characterize the performance of a predictive model, is processing Kvamme’s Gain index [44,45]. This index summarizes the model performance in a single value, relating the percentage of the total area covered by the model and the percentage of total sites within the model area (Eq 1). The output values range from -1 to +1, and higher index values indicate a better performance.

$$Kvamme\text{'}s\;Gain=1-\left(\frac{percentage\;of\;the\;total\;are\;a\;covered\;by\;the\;model}{percentage\;of\;total\;sites\;with\;in\;the\;model\;area}\right)$$ (1)

### 3.2. Spatial dataset of carbonate rock

The spatial dataset of carbonate rock distribution for our study region was produced by extracting polygons of surface and near-surface features containing carbonates of lithostratigraphic units of various ages, based on the ArcGIS platform developed by the Centre for Russian and Central EurAsian Mineral Studies’ (CERCAMS) ‘Mineral Deposits Database and Thematic Maps of Central Asia’ [28]. This material represents the first and only digital geological map of the Central Asia region that is available in the public domain. CERCAMS is continuously developing this geodatabase based upon own complex geoscientific studies, field tests and verification of formation ages using biostratigraphic and geochronological data, by updating its geological map that was initially developed out of the Soviet time 1:1,500,000 scale base map [46] and utilising the 1:200,000 geological maps and lithostratigraphic sections published by the Soviet Union Ministry of Geology until the late 1980s.

In using this dataset, we did not distinguish between carbonates of different ages, because before ground-truthing the model we preferred required not to rule out any carbonate-containing unit that may provide karstic conditions for cave formation. We must also assume some variation in the extents of the carbonate polygons, primarily because of the way extents for geologic units are inferred by geologists in the field.

Karstic landscapes produce a variety of distinctive morphologies, especially related to drainage patterns both ancient and modern. In our model, we were most interested in identifying areas where steep changes in topography might facilitate the exposure of carbonates on the vertical axis, either revealing entrances into pre-existing karstic systems or providing exposures for weathering processes to create negative features.

### 3.3. ASTER DEM

The developed models, described in detail in the following subsections (3.5–3.6), relied on the usage of the DEM of the Advanced Spaceborne Thermal Emission and Reflection Radiometer (ASTER). The ASTER ‘GDEM2’ was generated by using stereo-pair images, and a processed global DEM, ready for analyses. ASTER GDEM2 is a product of Japan’s Ministry of Economy, Trade, and Industry (METI) and NASA, and is freely available from NASA’S Land Processes Distributed Active Archive Center (*lpdaac*.*usgs*.*gov/products/astgtmv002*). The ASTER DEM offered full coverage of the study areas without seams or borders. Several DEM tiles of version 2.0 of the ASTER DEM were downloaded from the LP DAAC, and mosaiced in order to cover the combined extent of all study areas (Fig 1). After this operation, the DEM was projected to the Universal Transverse Mercator (UTM) system in Zone 44 North and using the World Geodetic System (WGS) 1984 ellipsoid (EPSG: 32644). The mosaic was finally resampled to a geometrical resolution of 35m by 35m, using the pixel aggregate function in the software ENVI 5.5 (*harrisgeospatial*.*com*) and elevation values were stored in floating point accuracy. The final DEM used in the analyses covered an area of approx. 2000km by 1100km. The ASTER DEM was chosen as it is of high precision, freely available, and offers higher spatial resolution than other freely available DEM products like the SRTM or the (free version of the) TanDEM-X DEM. High spatial resolution in turn provides better opportunity to distinguish appropriate features in the neighbourhood analysis, which provided the basis for both the 2018 and 2019 models.

### 3.4. Field surveys

Field surveys in the study area were conducted in 2017, 2018, and 2019. The PALAEOSILKROAD project conducted all field research under license No. 15008746 (12.05.2015) of the National Museum of the Republic of Kazakhstan based on the collaboration protocol between the Eberhard-Karls University of Tübingen and the National Museum. In 2017, basic exploratory survey was conducted in June and August. The majority of the 2017 survey was conducted in the Altai-Tarbagatai region. The 2017 survey was not guided by a model, but four cave and rockshelter features were located. The 2018 field survey was more intensive, and focused especially on the Qaratau range from May-June, followed by the Ili Alatau and Dzhungarian Alatau in August. The 2018 survey season was led by the first, unsupervised classification model, and located 73 cave and rockshelter features. This included a number of erosional hollows and funnels that are indicators of karst activity. These 77 features (from 2017 and 2018 combined) were included in the production of the 2019 supervised classification model. The 2019 survey was guided by the new, supervised classification model, and took place over May-June and August-September, and covered the Qaratau, Ili Alatau, and Altai-Tarbagatai areas. During this survey we identified an additional 28 cave and rockshelter features, for a current total of 105 features.

Prior to fieldwork, we developed a recording schema to complement the Paleo Core data structure developed by D. Reed (*paleocore*.*org*) [47,48], with the ultimate goal of integrating the results of our survey data into the PaleoCore system. Our goal is that the results of our survey and modelling will be widely available to our colleagues through open access. We implemented the recording schema through a series of customisable feature class forms in ‘GISpro’ (Garafa, LLC), a commercially available GIS app available for iOS, which were tailored to standardise input. An iPad Mini (Apple Inc.) was our primary data collection device in the field, using a Bad Elf GNSS surveyor (Bad Elf, LLC) for increased spatial accuracy in recording.

### 3.5. The 2018 model

**Fig 2:**
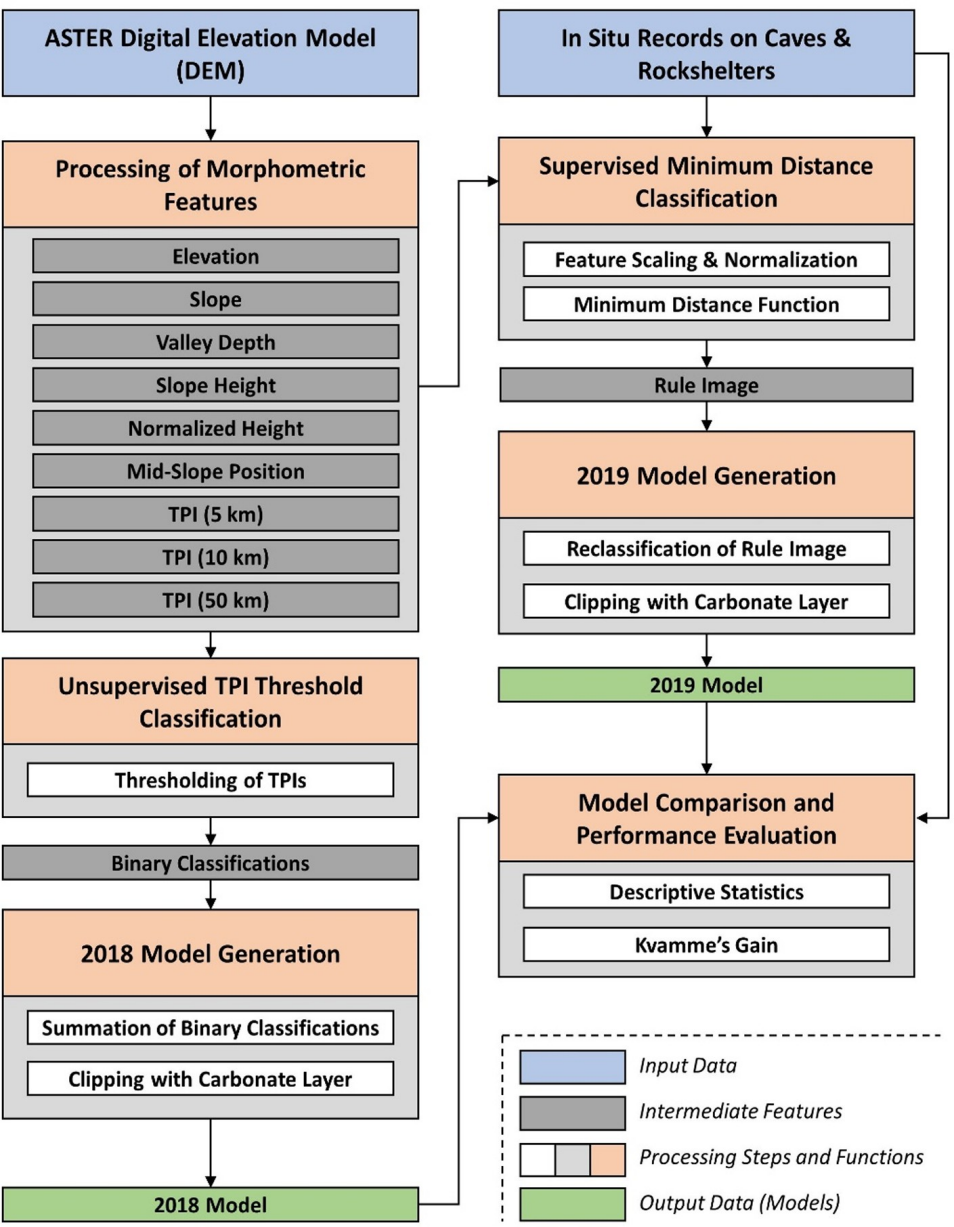
Schematic workflow on the generation of the two models (the ‘2018 model’ and the ‘2019 model’). The 2018 Model was generated without using any additional information besides the spatial distribution of carbonate rocks (“Carbonate Layer”), whereas the 2019 Model used *in situ* records on cave and rockshelter features to run a minimum distance classification approach.

The first model, subsequently referred to as the ‘2018 Model’, was generated by using morphometric features of the ASTER DEM in an unsupervised way (i.e. not using any information on the occurrence of rockshelter or cave features). The process of model construction is illustrated in Fig 2. The approach to classify topographic settings that might be indicative of the presence of rockshelters or caves was based on the concept of topographic position index (TPI) analyses [49,50]. While several alternative approaches for unsupervised landform classifications from DEMs exist (e.g. [51,52]), we chose TPI analysis for several reasons. First, TPI is an analysis that offers less intensive processing and intuitive interpretation compared to other geomorphometric features, such as topographic openness (e.g. [53]). These advantages render it a highly valuable, scale-related, feature for field interpretation and survey navigation. Processing complexity is a serious consideration due to the large size of the study area and the high resolution of the DEM (approx. 57000 pixels by 31000 pixels). Second, TPI quantifies the relative slope position of each pixel of the DEM with respect to a user-defined neighbourhood or scale. It is therefore an analysis that can be computed for several scales, allowing for multi-scalar analyses (e.g. [54]). Third, as TPI quantifies the relative slope position, it is appropriate for the identification of mid-slope positions. These, in turn, are believed to be most promising for the occurrence of caves and rockshelters [55]. In general, cave and rockshelters are unlikely to be detected in the present day at the foot-slopes of valley bottoms, due to the accumulation of soil material and/or scree released by hillslope processes. Furthermore, while locations up-slope might hold features of interest (especially rockshelters) these might have offered less sheltered (and therefore less-favoured) conditions for human occupation. Fourth, the successful application of TPI analyses in a (geo-)archaeological context has already been demonstrated to some extent in preliminary work (e.g. [56,57]).

TPI was processed using the ASTER DEM following Eq 1, where *x*_*i*_ is the elevation value of the pixel under observation, *MEAN* is the arithmetic mean elevation and *STDEV* the standard deviation of the elevation values in an estimation window centred over location *i*. The processing was done using the integral image approach [54], which was realized in the software IDL 8.7 (*harrisgeospatial.com*).

$$TPI_{i}=\frac{x_{i}-MEAN}{STDEV}$$ (2)

TPI is a normalized measure of slope position, where a TPI value of close to zero indicates that the pixel under observation is situated approx. at the mean elevation of the surrounding neighbourhood. Consequently, negative TPI values indicate valleys and foot slopes and positive TPI values indicate ridges and top-slopes [49,50,54]; however, the values depend on the size of the estimation window. The model was constructed by investigating three different scales using three different sizes of estimation window, which were 5km, 10km and 50km. Three TPIs were processed using estimation window sizes of 143 by 143 pixels, 287 by 287 pixels and 1429 by 1429 pixels. From Eq 1 it follows that correlation between TPIs of two consecutive scales increases with the size of the estimation window [54]. To balance the goals of the analysis with processing time and effort, only three scales were selected for the analyses, representing different slope positions in local (5km), regional (10km) and global (50km) context (see Table 1A).

**Table 1:** Investigated morphometric features: (a) features used in the 2018 Model and 2019 Model and (b) Correlation Matrix of the features (processed over the site locations (n = 77)). Values display the squared Pearson Correlation Coefficient (r²).

**(a) Investigated morphometric features**									
**Feature**	**Description**					**Unit**		**Model**	
Topographic Position Index (TPI)	relative slope position: normalized by the mean and standard deviation of a defined spatial neighbourhood (see Eq 1), TPIs were processed with scales of 5km, 10km and 50km					-		2018 & 2019	
Elevation	terrain surface elevation of ASTER DEM; meter above the WGS 1984 ellipsoid.					[m]		2019	
Slope	terrain slope in degree					[°]		2019	
Valley Depth	vertical offset in meter to closest modelled valley bottom					[m]		2019	
Slope Height	height in meter above the closest modelled drainage channels					[m]		2019	
Normalized Height	normalized difference between Slope Height and Valley Depth					-		2019	
Mid-Slope Position	index ranging from 0 to 1 indicating the slope position between minimum slope (0) and maximum vertical distances to valley bottom or ridge top (1)					-		2019	
**(b) Correlation Matrix–Squared Pearson Correlation Coefficient (r²)**									
	Elevation	Slope	TPI 5km	TPI 10km	TPI 50km	Valley Depth	Slope Height	Normalized Height	Mid-Slope Position
Elevation	1.00								
Slope	0.05	1.00							
TPI 5km	0.04	0.02	1.00						
TPI 10km	0.10	0.02	0.82	1.00					
TPI 50km	0.22	0.03	0.31	0.47	1.00				
Valley Depth	0.39	0.12	0.03	0.00	0.01	1.00			
Slope Height	0.06	0.16	0.22	0.35	0.42	0.00	1.00		
Normalized Height	0.04	0.02	0.42	0.28	0.18	0.38	0.34	1.00	
Mid-Slope Position	0.13	0.01	0.02	0.00	0.01	0.25	0.01	0.12	1.00

Features were processed using the ASTER DEM (35m by 35m spatial resolution). References for the feature processing and interpretation: [50,54,58,59].

Landform classification was performed at these different landscape scales, using the three different TPIs in the analysis. The identification of potential rockshelter or cave feature locations was thereby carried out by classifying the mid-slope positions from the TPIs. This was done by thresholding the TPIs with values ranging between -0.5 and +0.5, where this range is indicative for the mid-slope position [50]. The results of this operation were three binary classifications. These were summed up in a final classification system showing class values ranging from zero to three (0 = “none”, 1 = “low”, 2 = “medium” and 3 = “high”), where, for instance, a value of two indicated that TPIs of two scales fell within the defined range. This layer was clipped with the spatial dataset of carbonate rock, and the occurrence of classified pixels was deduced by converting the classification results to a point shape file and calculating the point density within in a radius of 10km. The point density was calculated to provide a quick overview on the model results at small scale. Both operations were carried out in ArcMap 10.6 (*desktop*.*arcgis*.*com*). The classification and the “heat map” layer served as a first orientation on the potential occurrence of carbonate rocks in mid-slope positions and was used in the first model-guided survey in 2018 to indicate most promising regions. The performance of the 2018 Model was evaluated by comparing the predicted class values with the locations where cave and rockshelter features were actually found in the field surveys in 2017, 2018 and 2019. Along with this, Kvamme’s Gain was processed.

### 3.6. The 2019 model

The second model, subsequently referred as the ‘2019 Model’, was constructed in a supervised way using results from the 2017–2018 field surveys (i.e. locations of caves and rockshelters that were documented during field work) and several morphometric features derived from the ASTER DEM in a supervised minimum distance approach [60]. The goal of the 2019 Model was twofold; firstly, we aimed to utilise our collected data on cave and rockshelter location to make predictions, and secondarily we aimed to increase the discrimination of the model to enable a more robust and focused approach to survey in the field.

The 2019 Model was constructed in the seven steps outlined below and in Fig 2.

1. The locations where caves and rockshelters were found in the 2017–2018 surveys (*n = 77*) were added to a common geodatabase in the Geographic Information System (GIS) ArcMap (desktop.arcgis.com).
2. The point locations of caves and rockshelters were buffered in the GIS using a radius of 200m. This was done to account for potential location inaccuracies and to allow an averaging of DEM features over the locations.
3. The morphometric features from the DEM TPI at the 5km scale, TPI at the 10km scale, and TPI at the 50km scale were processed in IDL. Additionally, the morphometric features terrain slope, Valley Depth, Slope Height, Normalized Height and Mid-Slope Position were processed in the software System for Automated Geoscientific Analyses (SAGA) (*saga-gis*.*org*) [61]. A summary of these features and their interpretation is provided in Table 1A and the assessment on the correlation among the features is presented in Table 1B. It should be noted that TPI 5km and TPI 10km revealed a strong positive correlation (r² of 0.82). Nevertheless, we decided to include both TPIs in the 2019 Model for the the sake of consistency in comparison to the 2018 Model, and as both features might still leave some potential for discrimination. Further details on the morphometric features are provided by Böhner & Selige [62], Dietrich and Böhner [58], and Kim et al. [59]. All investigated features have in common that they numerically describe the absolute or relative topographic setting or slope position by comparing the pixel value under observation to functional units (e.g. valley/ridge position, channel location, etc.) or constant spatial neighbourhoods (e.g. by using moving windows in the processing). While there are many other morphometric features that can be included in such an analysis, we have chosen the features listed in Table 1 as they can be processed rather quickly, provide normalized or standardized value ranges of the topographic setting, account for both functional and spatial units, and have been successfully applied in previous terrain and landform analyses (e.g. [58,59,62]).
4. The morphometric features were scaled to a common value range from 0 to 100 using ENVI 5.5, the “Stretch Data” function, floating point accuracy and a lower threshold of 0.5% and an upper threshold 99.5% for the linear stretch, e.g. a value of 100 then indicates the feature value at the 99.5% percentile. The “Stretch Data” function allows comparing the morphometric features on a common value range, which is a perquisite for the following minimum distance classification.
5. ENVI’s “Minimum Distance” function (see [60]) was applied by using the buffered cave and rockshelter locations and the stack of all scaled morphometric features (Table 1). The features were considered equally significant for the identification of caves/rockshelters, as such they contribute in the same way to the model outputs. The usage of additional threshold was disabled, but the rule image was generated and used in further analyses. The rule image displays the Euclidean distance from the class mean vector, i.e. low values indicate pixels that share similar morphometric properties with the feature values of the known cave and rockshelter locations. The distance is measured in the same unit as the input variables, e.g., a distance of 10 indicates that the mean distance between the feature values of the rockshelter and cave locations was less than 10% of the value range of the feature, as all features were scaled to values from 0 to 100 using the 0.5% and 99.5% percentiles. In this way, the rule image predicts similar topographic situations with higher and lower likelihood of containing similar features.
6. The rule image was classified in four classes (0 = “none”, 1 = “low”, 2 = “medium” and 3 = “high”), by applying thresholds of > 50% = “none”, 50% to 30% = “low”, 30% to 10% = “medium” and <10% = “high” to the rule image.
7. The classification result was clipped to the extent of the carbonate layers.

This classification served as an orientation toward potential locations that share topographic characteristics that are similar to the locations of our already discovered features. It was used in the second model-guided field survey in 2019. The performance of the 2019 Model was evaluated by comparing the predicted class values with the locations where cave and rockshelter features were actually found in the field surveys 2017, 2018 and 2019. This means that the test shows how good the model is in self-predicting the input features. However, as mostly the same reference data were used to conduct the minimum distance approach (77 out of the 105 records were used to construct the model), the evaluation is not fully independent. Further, it has to be assumed that the found sites are representative of the actual variance of caves/rockshelter locations. Nevertheless, such an analysis allows assessment of the consistency of the reference data, by roughly evaluating the ‘fit’ of the reference to data to the model produced from it. If the features recorded *in situ* are located in a similar morphometric context, they will be characterized by similar values in the rule image and the classification. If not, this assessment will indicate that a simple minimum distance approach is not applicable for the problem, at least not from the available samples. Beside this, the model performance is evaluated by using the Kvamme’s Gain index.

## 4. Results

### 4.1. The 2018 model

**Fig 3:**
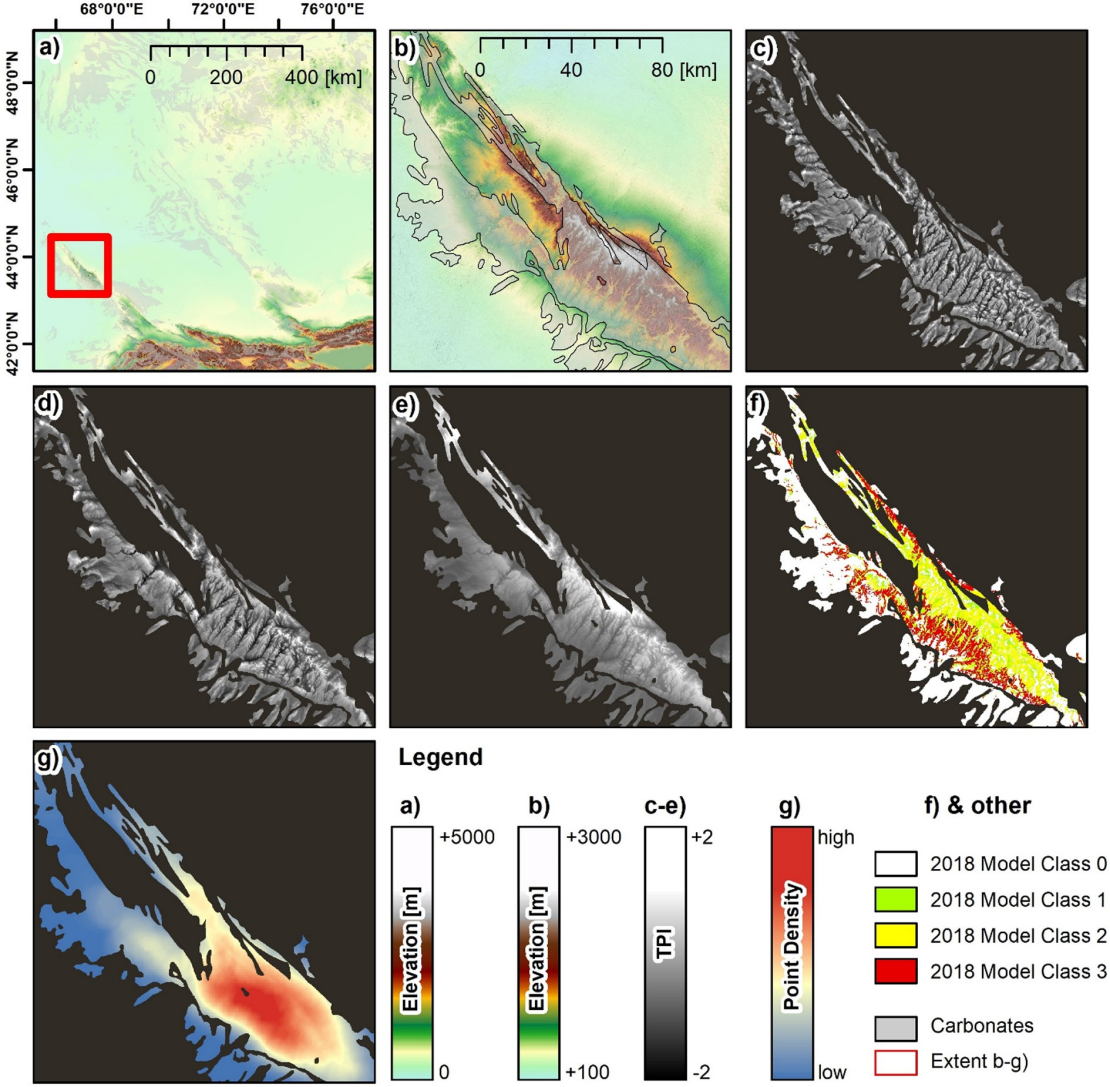
Example of the 2018 model. (a) ASTER DEM of the study area and spatial extent of carbonate rock, (b) ASTER DEM and spatial extent of carbonate rock of the Qaratau mountain range, (c) Topographic Position Index (TPI) processed at a scale of 5km, (d) TPI processed at a scale of 10km, (e) TPI processed at a scale of 50km, (f) classification result of the 2018 Model and (g) point density of class occurrence with in a search radius of 10km. UTM Zone 44N, WGS 1984 ellipsoid (EPSG: 32644) Contains data from ASTER GDEM2 (see section 3.4 for full information). f shows the classification result of the 2018 Model (i.e. the classification of the TPIs for the value range -0.5 to +0.5 and the resulting overlay). Particularly, Class 3 shows a clear pattern. The class locations constitute a stretched belt along the southern flank in the mid-slopes of the Qaratau range (due to TPI values at 50km scale) and at heads and middle courses of the main valleys (due to the TPI values at 10km scale). This is as well highlighted by the point density of class occurrence in g. This layer indicates a high point density for the southern mid-slopes of the Qaratau range, while point density is lower for the northern part of the range and the southern escarpment outliers that are situated between the northern uplands and the southern lowlands. Note in this context that point density is sensitive to the masked non-carbonate locations (i.e. these do not account towards the density).4.2. The 2019 model.

Fig 3 highlights the results of 2018 Model for the Qaratau mountain range. As indicated, the model construction relied solely on the classification of three TPIs, processed at scales of 5km (Fig 3C), 10km (Fig 3D) and 50km (Fig 3E). The TPIs highlighted the configuration of landforms at different scales, at the respective varied landform sizes. TPI values at the lowest scale (5km) indicate local small valleys and smaller landform features within a valley. TPI values therefore vary largely at short distance and highlight the local landform setting and the variation of the slope position on a small scale, respectively. The 10km scale TPIs highlight the configuration of landforms on the regional scale. For instance, the TPI indicates the northwest to southeast oriented ridges in the central part of the Qaratau mountain range, as well as several valley systems. TPI variations take place less frequently over short distance. The 50km scale TPI highlights the relative slope positions within the entire Qaratau mountain range and this feature indicates the overall slope position within the range.

**Fig 4:**
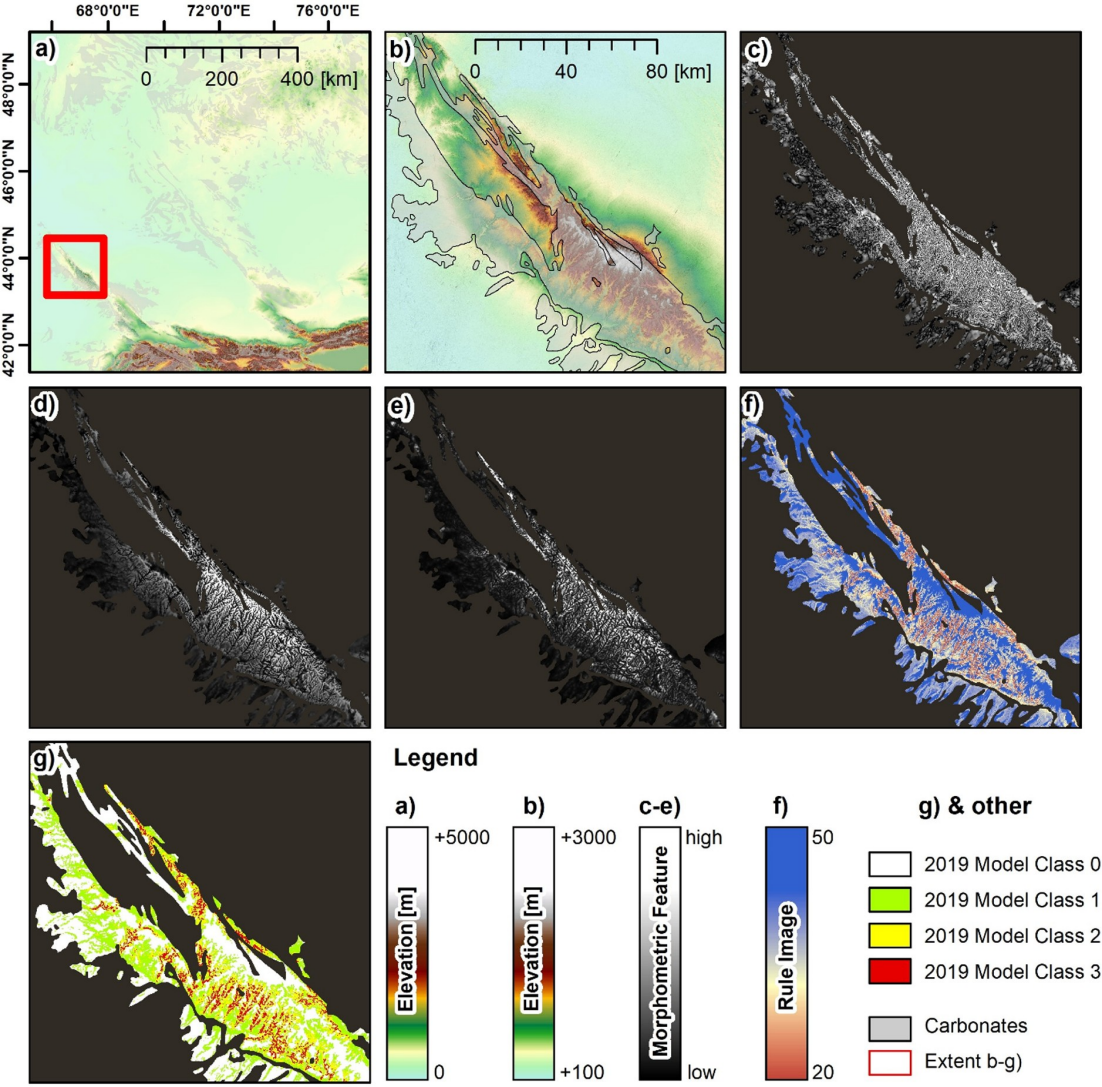
Example of the 2019 model. (a) ASTER DEM of the study area and spatial extent of carbonate rock, (b) ASTER DEM and spatial extent of carbonate rock of the Qaratau mountain range, (c) morphometric feature “Valley Depth”, (d) morphometric feature “Standardized Height”, (e) morphometric feature “Slope Height”, (f) rule image of the minimum distance approach trained using *in situ* records on the occurrence of caves and rockshelters and (g) final classification result of the 2019 Model. UTM Zone 44N, WGS 1984 ellipsoid (EPSG: 32644) Contains data from ASTER GDEM2 (see section 3.4 for full information).

Fig 4 shows the results of the 2019 Model for the example of the Qaratau mountain range. The model was generated using a minimum distance classification (Section 3.6), the locations of *in situ* recorded caves and rockshelters, and the morphometric features listed in Table 1. Among the morphometric features used in the classification, the figure shows examples of Valley Depth (Fig 4C), Standardized Height (Fig 4D) and Slope Height (Fig 4E). These features are sensitive to small landform elements, and therefore account primarily for the local and regional setting, rather than the overall topographic setting of the mountain range. Standardized Height clearly highlights the valley-ridge sequences of the southern flank, whereas the Valley Depth feature indicates more deeply-incised valleys in the mid-position of the range, compared to the valleys of the northern part of the range and the southern escarpment outliers. Similarly, the Slope Height feature is higher for valleys in the mid-position of the range, indicating a steeper gradient and higher vertical offsets of the valley flanks to the valley bottom, in the drainage channels and erosion lines respectively. Fig 4F shows the rule image of the minimum distance classification that was processed using all of the morphometric features (Table 1) and the *in situ* recorded locations of caves and rockshelters.

The lowest distances between the ‘morphometric signature’ of the *in situ* records and the morphometric setting of the Qaratau mountain range are found along the southern flank of the range, in mid-slope positions and along the flanks of the incised valleys in the more central part of the range. The rule image clearly indicates that valley bottoms have a less similar signature (i.e. higher distance in the rule image), which is reasonable as *in situ* finds were most frequently located in the mid-slopes and not in the bottoms of the valley systems; a fact that is captured by the 2019 Model. The lowlands of the outliers and the highlands towards the central summits of the range occur with greater distance in the rule image and are therefore indicated to have less similar morphometric settings compared to the *in situ* record. Similarly, the northern mountain range is indicated to have a different setting, compared to the morphological situation that was found for the *in situ* records. Fig 4G shows the final classification map that was produced by applying the thresholds indicated in Section 3.6 to the rule image. The strict constraint for Class 3 (= average deviation from the *in situ* records in the rule image less than 10%) results in very few isolated locations that are predominantly found in the mid-slopes of the southern valleys of the range. These locations are surrounded by locations of Class 2, which is also the class that most frequently occurs in the southern part of the mountain range. Class 1 covers the more northern parts of the range and outliers of the southern escarpment.

### 4.3. Model comparison and evaluation

Comparing the two models, the coverage remains the same (clipped to the carbonate layer), but the discrimination increased between iterations. This can be seen most clearly in the change in area for the model’s low (Class 1), medium (Class 2), and especially the high (Class 3) predictive values within the focus area of the IAMC (see Table 2). Whereas Class 3 accounts for around 30% of the 2018 model’s area, this is reduced to 7% of the total in the 2019 model. The changes between categories are less important than the total change of predictive value between the models, which can be seen in Table 2.

**Table 2:** Classified areas within the focus area of the IAMC covered by the 2018 and 2019 models in km^2^, including distribution by Class and change in % between iterations of the models.

Predictive Value	2018 Model	2019 Model	% Difference in Area (from 2018 to 2019)
Class1	7,066km2	11,595km2	+64.1%
Class2	6,977km2	4,520km2	-35.2%
Class3	5,957km2	1,130km2	-81.0%
**Total**	**20,000km2**	**17,245km2**	**-13.7%**

In practice, the increase in discrimination between the two models allowed us to focus our survey on areas and landforms that were more likely to yield results. As an area of the IAMC, the 2019 model represents a narrowing of the focus down to around 5% of the total area of the IAMC within Kazakhstan, in comparison to 12% in the 2018 model.

**Fig 5:**
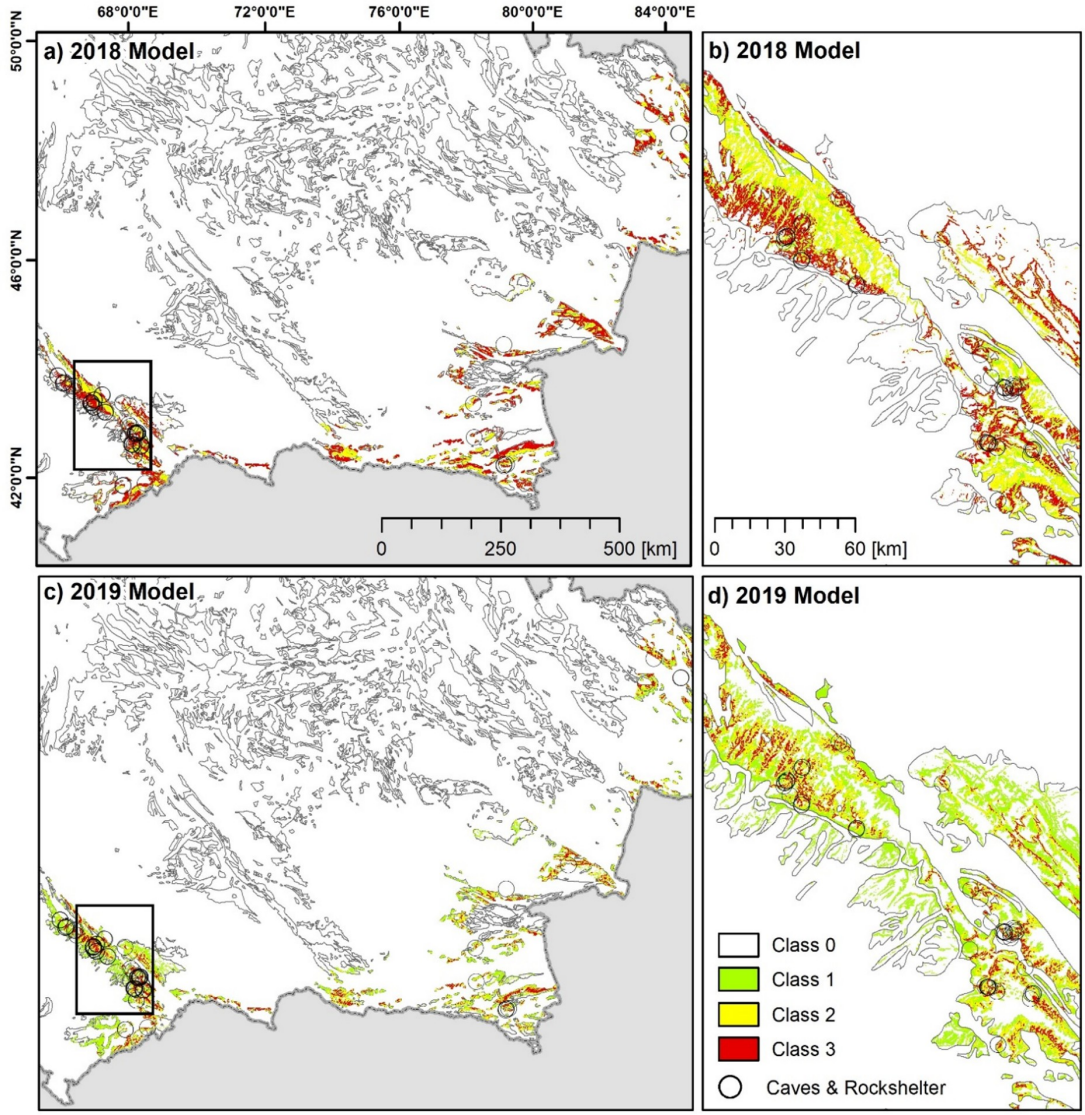
Comparison of the 2018 model and the 2019 model. (a-b) the 2018 Model and (c-d) the 2019 Model. Enlargement of the models focus on the central Qaratau mountain range. *In situ* records of caves and rockshelters are indicated by pink circles. UTM Zone 44N, WGS 1984 ellipsoid (EPSG: 32644) Contains data derived from ASTER GDEM2 (see section 3.4 for full information). Administrative boundaries use copyrighted map data from OpenStreetMap contributors [29], available from openstreetmap.org.

Fig 5 shows results of both models for the entire study region and for a selected subset with more spatial detail. The comparison shows that higher point density and class numbers of both models are generally found in the four selected key study regions, which means that both models predict a high chance of cave and rockshelter occurrence for regions with significant topography and relief energy respectively. This suggests that carbonate rock locations in the lowlands have a lower chance of cave and rockshelter occurrence.

The 2018 model provides more general information with less spatial detail compared to the 2019 model (compare Fig 5E and 5F). Entire mountain ranges instead of individual locations are indicated. For example, large parts of the Dzhungarian Alatau are characterized by high point densities (Fig 5A), which does not allow for singling out specific locations, such as individual valleys, for investigation. However, the 2018 model does provide a first orientation in which model-guided regional field survey might be more efficient and targeted.

**Fig 6:**
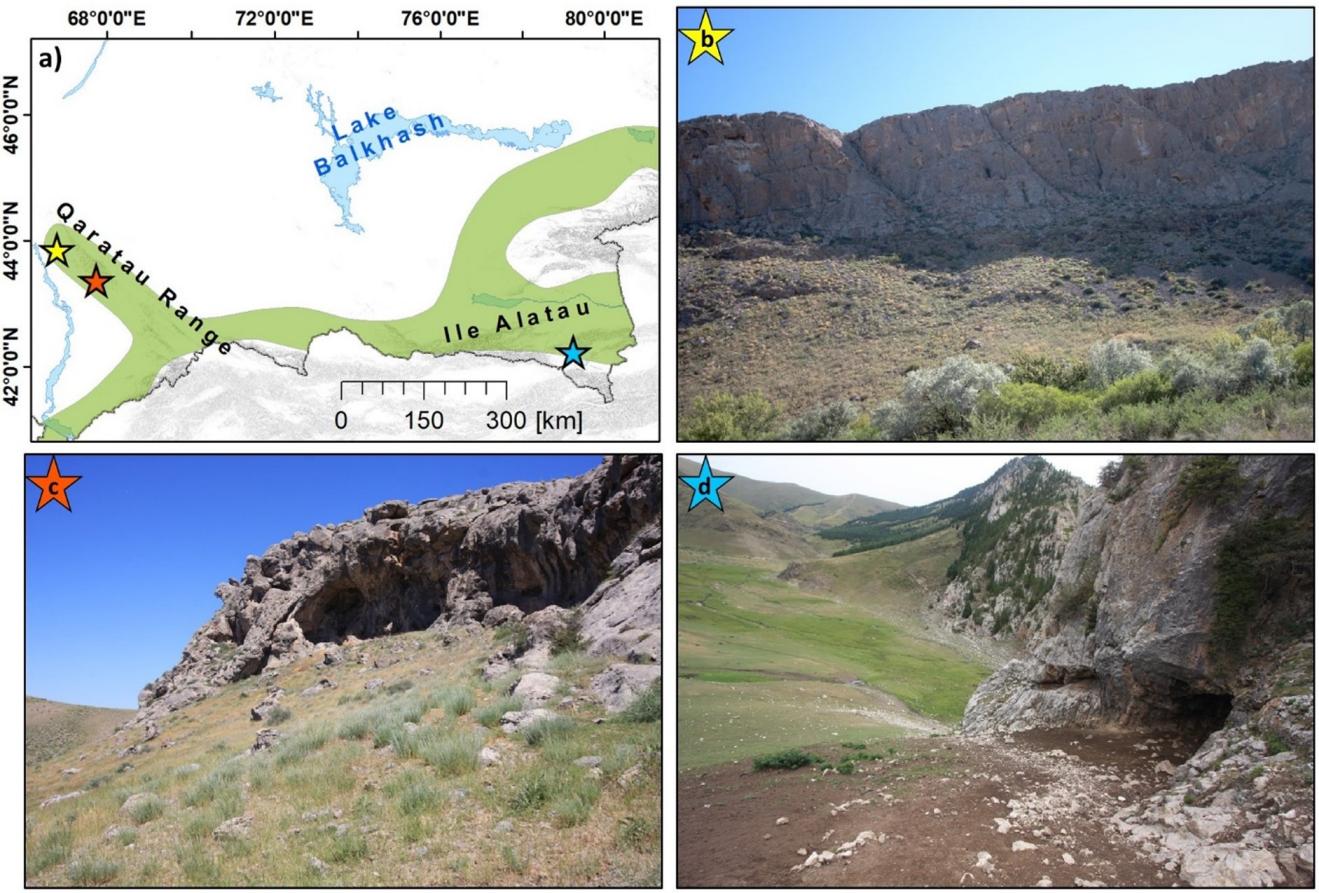
Examples caves and rockshelter features. A) Aquiq 1 cave. Inaccessible cave formed along vertical joints. Minor karstic features like the crevices and hollows that are ubiquitous all over this particular cliff face were not recorded as individual features, but as one collective feature. B) Qyzkorgan 3 rockshelter. Features wider than deeper like Qyzkorgan 3 were identified as ’rockshelters. C) Aqtasty 3 cave. We identified caves as features deeper than they are wide.

The 2019 Model provides higher spatial detail due to the model construction and the morphometric features used. Fig 5C and 5D highlight the model outputs for the Qaratau mountain range and indicate specific locations that show the best match to the topographic setting of the discovered locations. As mentioned in the preceding section, locations with the smallest deviation from the *in situ* record are found in the mid-slope positions of valleys and in the central part of the mountain range. Fig 6 shows examples of karstic features, including caves and a rockshelter, which were identified during survey.

**Fig 7:**
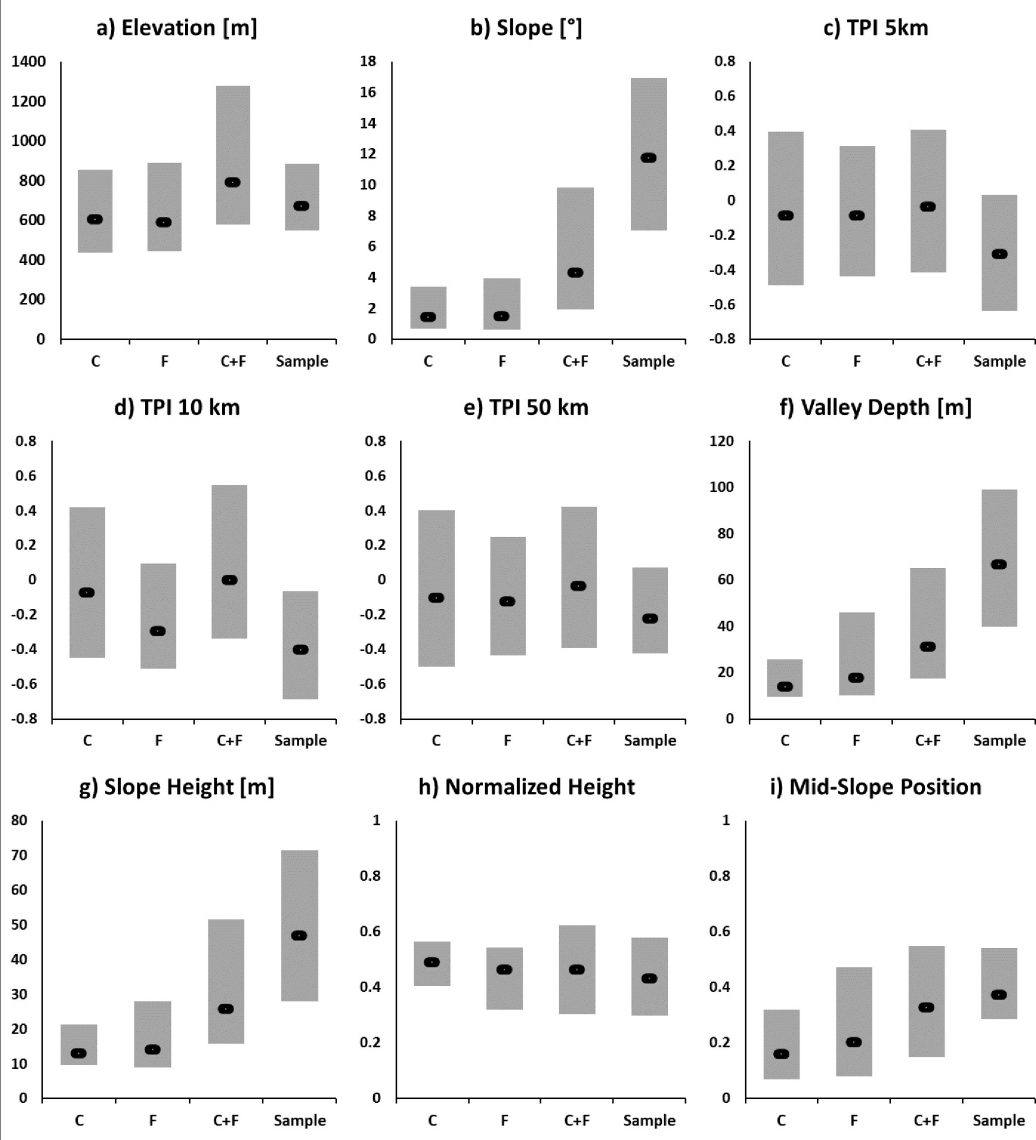
Descriptive statistics of the morphometric features. (a) Elevation, (b) Slope, (c) Topographic Position Index (TPI) processed at a scale of 5km, (d) TPI processed at a scale of 10km, (e) TPI processed at a scale of 50km, (f) Valley Depth, (g) Slope Height, (h) Normalized Height and (i) Mid-Slope Position. Bars indicate the inter-quartile-range (IQR) between the 25% and the 75% percentiles. The black marker indicates the position of the median (50% percentile). Statistics are drawn for; “C” (yellow) = the carbonate layer (approx. 214km²), “F” (blue) = the focus area indicated in Fig 1 (approx. 209km²), “C+F” (green) = carbonate layer inside the focus area (approx. 32km²), “Sample” (red) = location of *in situ* records on Caves and Rockshelters. Stats are based on the records found during the 2017 and 2018 field survey (*n = 77)*.

The topographic signature provided by the *in situ* records has been further analysed in order to better understand and quantify the morphological settings that are indicative of cave and rockshelter locations. Fig 7 shows descriptive statistics of the *in situ* records for the morphometric features we utilised (Table 1) in comparison to the statistics of carbonate layer, the study areas, and the combined extent of the carbonate layer and the study areas. This analysis therefore accounts for the statistical difference between the sample (caves and rockshelters in carbonate rock) and the entire population (all locations of carbonate rock, of the study areas, and the combined extent). This comparison revealed, in descending order of significance indicated by the separation of the boxes of the interquartile ranges (IQR, i.e. the range between the 25% and the 75% percentiles) that cave and rockshelters are situated (i) mostly in steep terrain (Fig 7B; IQR of the terrain slope ranging between 6° to 16°), (ii) at positions with significantly higher Valley Depths (Fig 7F; IQR ranging from 40m to 100m) and Slope Heights (Fig 7G; IQR ranging from 25m to 70m), and (iii) at intermediate Mid-Slope-Positions (Fig 7I; IQR ranging from 0.30 to 0.55). IQR overlap between the sample and the other populations (C, F, C+F) is rather large for the other morphometric features, and these features are therefore less indicative for the sample as they share the common characteristics of all carbonate rock locations in the study areas. Among these features of lesser importance, the TPI features were indicated by negative mean TPI values around -0.4 and IQRs of approx. -0.55 to 0.0, which is an indicative range for mid-slopes at the transition to the foot-slope and/or for local depressions.

**Fig 8:**
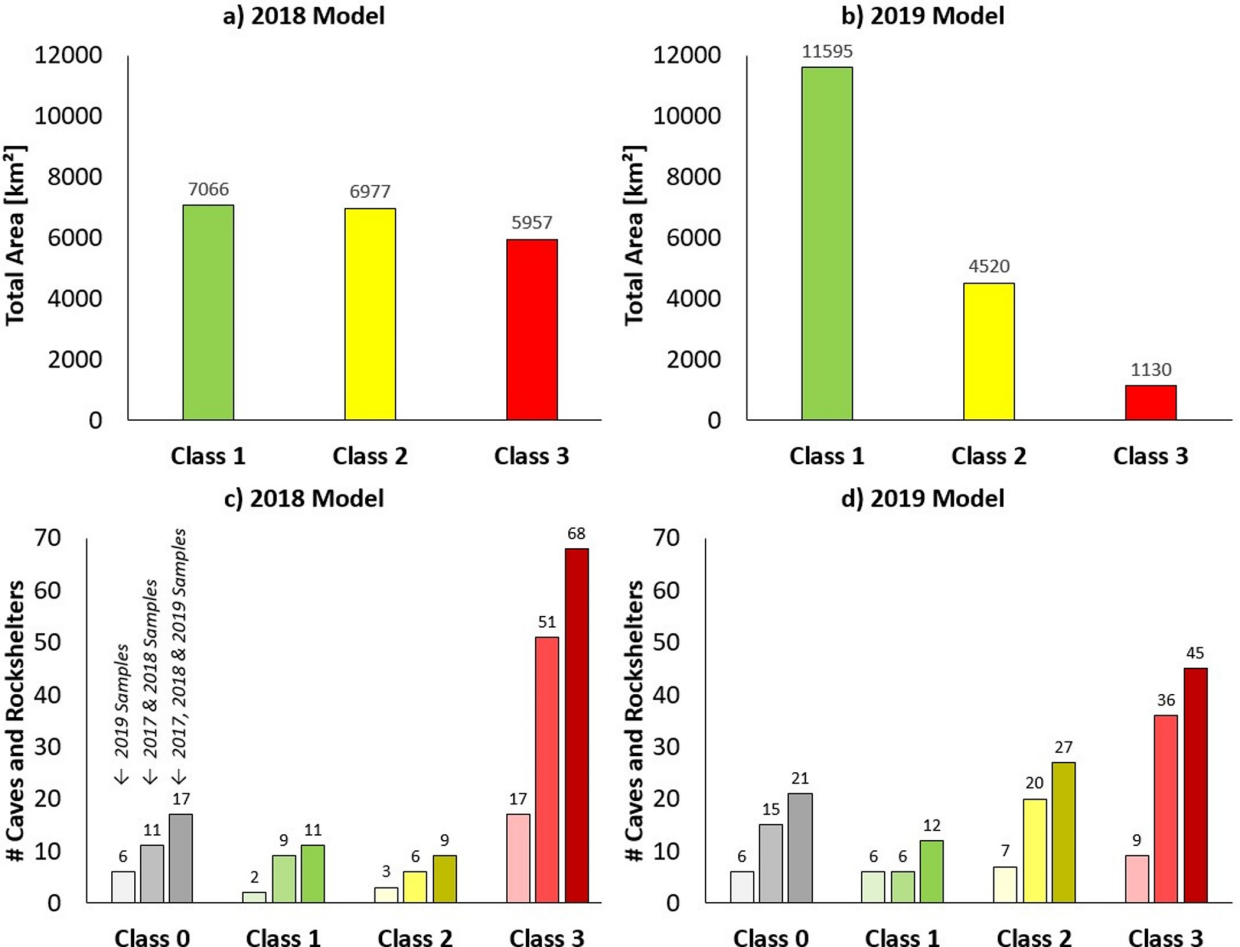
Evaluation of the 2018 model and the 2019 model. (a) Total area of the classes in the 2018 Model, (b) Total area of the classes in the 2019 Model, (c) number of *in situ* recorded caves and rockshelters per class of the 2018 Model and (d) number of *in situ* recorded caves and rockshelters per class of the 2019 Model. In (c) and (d) the used sample is indicated: (i) features found in 2019 using the 2019 Model (*n = 28*), (ii) features found in 2017 and 2018 (*n = 77*) and (iii) all features found in the 2017, 2018 and 2019 surveys (*n = 105*).

The performance of the 2018 model and the 2019 model is shown in Fig 8 and Table 3. Fig 8A and 8B show the total area that is covered by the individual classes. For the 2018 model these data underline that the classification is not very specific, but the occurrence of Class 1, Class 2, and Class 3 is–more or less–distributed equally. The 2019 model demonstrates stricter constrains for the classification and therefore the total area significantly decreases from Class 1 to Class 2 to Class 3, which narrows done the prospective area for field survey. Fig 8C and 8D show how the observed cave and rockshelter locations relate to the two classifications. For the 2018 model, it was found that most of the records are classified as Class 3 (= 68), while 20 records belonged to Class 2 or Class 1. A total of 17 records fall outside the classification range (Class 0). For the 2019 model, 45 locations are in Class 3, while 27 locations are in Class 2. Class 1 shows 12 records, and 21 records fall outside the classification range (Class 0). For the 2019 model, this evaluation indicates the capacity of the model to self-predict the reference data that were used to construct the model. This means that the evaluation shown in Fig 8 is not independent; the assessment rather evaluates if the applied minimum distance approach is reasonable and applicable. It shows that even though the total area of Class 2 and Class 3 is small (<2000km²), the number of *in situ* classes that are assigned to these is classes is very high (= 72 in total).

**Table 3:** Comparison of the models via Kvamme’s Gain for (a) the 2018 Model and (b) the 2019 Model using all available samples from the 2017, 2018 and 2019 surveys (*n = 105*). The percentage area is processed relative to the carbonate layer inside the focus area indicated in Fig 1.

**a) 2018 Model**			
	Percentage Area [%]	Percentage Sites [%]	Kvamme’s Gain
Class 3	6.55	64.76	+0.90
Classes (2 & 3)	14.20	73.33	+0.81
Classes (1, 2 & 3)	21.94	83.81	+0.74
**b) 2019 Model**			
	Percentage Area [%]	Percentage Sites [%]	Kvamme’s Gain
Class 3	1.24	42.86	+0.97
Classes (2 & 3)	6.19	68.57	+0.91
Classes (1, 2 & 3)	18.91	80.00	+0.76

Finally, Table 3 presents the results of Kvamme’s Gain for the 2018 and the 2019 model. The values are strongly positive and the assessment allows comparing the performance among the different models and their classifications. Results support the previous stated findings. The 2019 Model allows significantly reducing the target area, while keeping a high number of site records.

## 5. Discussion

The 2018 model was an unsupervised form of classification model, and this allowed us to open up a wide area for survey, targeting aspects of the physical environment that we reasoned from the literature and direct observation would have an impact on cave formation. The 2019 model, in contrast, relied on a supervised minimum distance approach, and therefore aimed to understand the geomorphic situation of features we had already found, and to extend this across the study region for increased discrimination. Having surveyed in all of our study areas by the time we developed the 2019 model, we had amassed a good and representative sample of existing cave and rockshelter features from a variety of geomorphic situations. In this way, we were not limiting our search to an artificial subset of caves and rockshelters. This is supported by the boxplot statistics in Fig 7, which show rather small IQRs for all of the features, and therefore demonstrates fairly common characteristics among the found feature locations. This enabled us to use the 2019 model to reduce the total survey area and focus our survey on areas likely to provide features that met our search criteria more accurately than in the first iteration of the model.

While more intensive supervised modelling techniques exist, we consider the use of the minimum distance approach for the 2019 Model as appropriate, effective, and practical here, as it showed a good performance indicating potential sites, as well, it allows for a intuitive interpretation of the results, which is advantageous considering the usage during fieldwork. This considerably improved the field navigation and survey performance and enabled a better rate of cave detection, and also increased the quantity and quality of the yielded ground-truth-data.

Even though it cannot be done fully independently of the data used to produce it, the evaluation of the 2019 Model revealed that the rather simple minimum distance approach is capable of predicting most of the *in situ*, validated locations with a high precision. For instance, 72 out of 105 records were assigned to Class 2 or Class 3, when using all available records (2017, 2018, and 2019) for the performance evaluation. Further, index values of Kvamme’s Gain were highly positive (+0.91). This high level of performance can be explained by the indicative and distinct value ranges provided by some morphometric features for the cave and rockshelter locations (see Fig 7 in this context). The boxplot statistics revealed that the sample locations of cave and rockshelter features in carbonate rock is, for some features, considerably different to the entire population (i.e. all locations possible in carbonate rock areas). This helps to narrow down the ground survey to target locations that show such indicative morphological settings. In summary, a rather large topographic gradient (terrain slope of approx. 6° to 16°), a relative slope position at the transition between the mid- and the foot-slope, as well as, Valley and Slope Heights between 40m and 100m seem to be promising terrain characteristics that are indicative features for future surveys. This suggests that future work to identify cave and rockshelter features in Kazakhstan should continue to target mountainous terrain, as exemplified by our four key study areas and the area of the IAMC.

However, three main limitations of the chosen approach must be noted. Firstly, the quality of the data inputs have a direct impact on the quality of the model. The data science maxim of ‘Garbage In Garbage Out’ applies just as much to model-building [63], where the model can only be as good as the lowest quality dataset. Rather than being mitigated in the process of combination with better datasets, the issues with problem datasets are exacerbated and cascade through the process of model-building. Where possible, all data used in such models need to be of a known quality, and ground-truthing field survey is invaluable for providing such feedback. Furthermore, the results of models should be evaluated where possible, either through independent means, or to show that they are at least internally consistent with the data used to produce them, as we demonstrated with the 2019 model.

Secondly, only one class is targeted and therefore the event occurrence (caves and rockshelters) cannot be compared with non-event occurrence. Furthermore, it is clear that the probability of the existence of a cave or rockshelter feature is much lower than the probability of its absence, but this *a priori* probability cannot be derived from the current *in situ* samples.

Thirdly, a drawback of the minimum distance approach is that non-linear relationships might not be detected, as only the Euclidean distance is investigated in such an analysis. We consider this issue only of minor relevance to the present study, as the main objective of the model is to guide field survey, and therefore the model aims to indicate where caves and rockshelters are generally to be expected, and not to predict single caves or rockshelters for individual topographic situations or further site characteristics. However, future work will also consider such non-linear relationships that, for example, might be present due to the different genesis of the features, or as features are situated in specific rock formations. In turn, the presence of sub-classes might be uncovered in the statistics of the morphometric once the database of *in situ* validated cave and rockshelter locations is increased by further field survey. New means, to assess sub-classes in upcoming work, will be the use of non-parametric classifiers (e.g. the Random Forest approach [64], which would also allow investigating the variable importance), and that have also been applied in related work [27].

## 6. Conclusion

The PALAEOSILKROAD project has spent two years building and ground-truthing models for karstic cave prediction in our study regions in the mountainous areas of Kazakhstan. Our goal was to locate and study new cave and rockshelter features in the region. Over this time period we have surveyed 105 cave and rockshelter features in the study region, around 30% of which have some amount of accumulated sediment.

Our first model was built with an unsupervised landform classification derived from an ASTER DEM of our study region, which was then clipped to the extent of surveyed carbonates in the region. We used this model to lead survey in the 2018 field season, where we identified 73 cave and rockshelter features. We concluded that the model was correctly identifying large areas of the landscape that could contain karstic caves and rockshelters, but we also hoped to increase the discrimination of the model further, and thereby reduce the survey area.

Our second model was built using a supervised minimum distance approach, utilising location data of cave and rockshelter features identified in the 2018 survey as well as morphometric features derived from the ASTER DEM. This model identified areas that were topographically similar to locations where cave and rockshelter features had been identified during the 2018 survey season. We achieved an increase in discrimination between the two models to allow more targeted field survey. The 2019 model in particular highlighted the importance of steep terrain, high valley depth, high slope height, and intermediate mid-slope position as key morphometric features for predicting cave and rockshelter features.

The simplicity of these models, relying as they do on only the extent of formations containing carbonate rock and landform classification on freely available DEMs, means that they are in principle possible to replicate anywhere that such data exists.

Although ground-truthing is often difficult and field survey is beset by logistical and scientific obstacles, we affirm its importance for the continued development of predictive models, and also the value of model-guided field survey in overcoming these obstacles. In particular, the use of both unsupervised and supervised classification methods can allow a flexible approach, the former opens the area for analysis, and the latter can help extend and increase discrimination to discover similar situations elsewhere, and begin to identify the factors that determine relevant feature location.

In the future, we plan to investigate the factors that lead to the accumulation of archaeological sediments in caves. An additional avenue of research will explore the relationships within subsets of the cave and rockshelter features, for instance, by age of the parent rock, by morphological attributes of the features themselves, or in context with geological peculiarities such as faults or bedding.

## Supporting information

S1 File (TXT)
