# Correction: Finding karstic caves and rockshelters in the Inner Asian mountain corridor using predictive modelling and field survey

**DOI:** 10.1371/journal.pone.0250142

**Published:** 2021-04-08

**Authors:** Patrick Cuthbertson, Tobias Ullmann, Christian Büdel, Aristeidis Varis, Abay Namen, Reimar Seltmann, Denné Reed, Zhaken Taimagambetov, Radu Iovita

The caption for Fig 6 is incorrect. Please see the complete, correct Fig 6 caption here.

**Fig 6:**
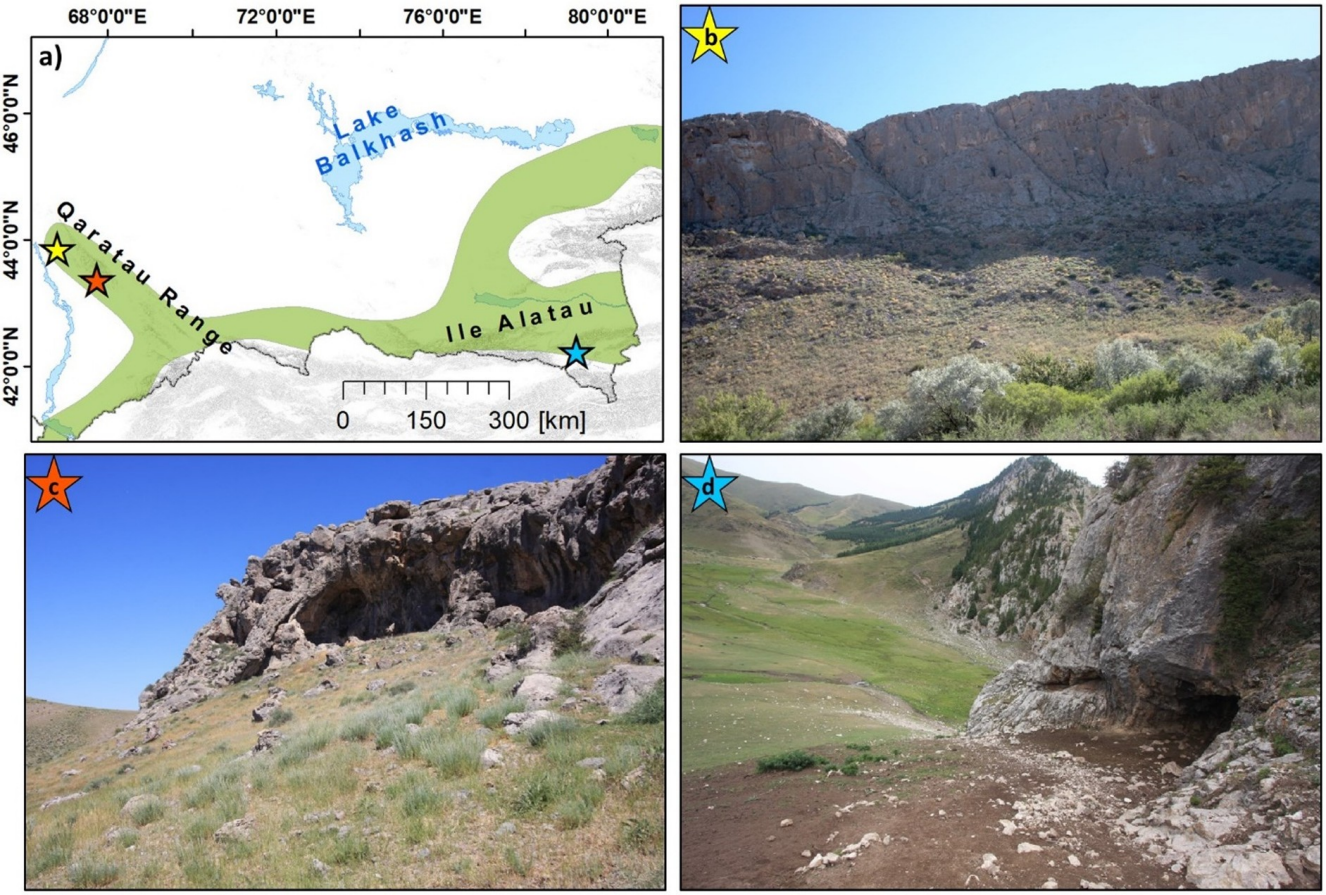
Examples caves and rockshelter features. A) shows an overview of the IAMC in South Kazakhstan with the locations of the highlighted karstic features. B) Aquiq 1 cave. Inaccessible cave formed along vertical joints. Minor karstic features like the crevices and hollows that are ubiquitous all over this particular cliff face were not recorded as individual features, but as one collective feature. C) Qyzkorgan 3 rockshelter. Features wider than deeper like Qyzkorgan 3 were identified as ‘rockshelters. D) Aqtasty 3 cave. We identified caves as features deeper than they are wide.

## References

[1] Cuthbertson P, Ullmann T, Büdel C, Varis A, Namen A, Seltmann R, et al. (2021) Finding karstic caves and rockshelters in the Inner Asian mountain corridor using predictive modelling and field survey. PLoS ONE 16(1): e0245170. 10.1371/journal.pone.0245170 33471843 PMC7816991

